# Decreased MARCKS Protein Expression in Kidney Cortex Membrane Fractions of Cathepsin B Knockout Mice Is Associated with Reduced Lysophosphatidylcholine and Protein Kinase C Activity

**DOI:** 10.3390/biomedicines11051489

**Published:** 2023-05-20

**Authors:** Tamim Kawakibi, Niharika Bala, Lauren P. Liu, Louis A. Searcy, Nancy D. Denslow, Abdel A. Alli

**Affiliations:** 1 Department of Physiology and Aging, University of Florida College of Medicine, Gainesville, FL 32610, USA; 2 Department of Physiological Sciences and Center for Environmental and Human Toxicology, University of Florida College of Veterinary Medicine, Gainesville, FL 32610, USA; 3 Department of Medicine, Division of Nephrology, Hypertension, and Renal Transplantation, University of Florida College of Medicine, Gainesville, FL 32610, USA

**Keywords:** MARCKS, cathepsin B, lipids, protein kinase C

## Abstract

Cathpesin B is a multi-functional protease that plays numerous roles in physiology and pathophysiology. We hypothesized that actin cytoskeleton proteins that are substrates of cathepsin B, various lipids, and kinases that are regulated by lipids would be down-regulated in the kidney of cathepsin B knockout mice. Here, we show by Western blot and densitometric analysis that the expression and proteolysis of the actin cytoskeleton proteins myristoylated alanine-rich C-kinase substrate (MARCKS) and spectrin are significantly reduced in kidney cortex membrane fractions of cathepsin B knockout mice compared to C57B6 wild-type control mice. Lipidomic results show that specific lipids are increased while other lipids, including lysophosphatidylcholine (LPC) species LPC (16:0), LPC (18:0), LPC (18:1), and LPC (18:2), are significantly decreased in membrane fractions of the kidney cortex from Cathepsin B null mice. Protein Kinase C (PKC) activity is significantly lower in the kidney cortex of cathepsin B knockout mice compared to wild-type mice, while calcium/calmodulin-dependent protein kinase II (CaMKII) activity and phospholipase D (PLD) activity are comparable between the two groups. Together, these results provide the first evidence of altered actin cytoskeleton organization, membrane lipid composition, and PKC activity in the kidneys of mice lacking cathepsin B.

## 1. Introduction

The multi-functional cysteine protease cathepsin B is known to play a role in several biological processes including ferroptosis [1,2], tumor cell proliferation and metastasis [3], lipid metabolism [4], inflammasome activation [5], activation of proenzymes [6], and activation of ion channels [7,8]. Cathepsin B is packaged in extracellular vesicles [9] and also released into biological fluids [10,11]. The actions of cathepsin B are not limited to intracellular activity but it can act in a paracrine dependent manner.

Cathepsin B plays important roles in physiology and pathophysiology in several organs including the kidney. In the distal nephron and collecting duct of the kidney, cathepsin B is involved in the proteolysis of a myriad of proteins including the epithelial sodium channel (ENaC) [7,8] and the multifunctional actin cytoskeleton protein myristoylated alanine-rich C-kinase substrate (MARCKS) [12]. MARCKS positively regulates ENaC in a PIP2-dependent manner in polarized renal epithelial cells of the distal nephron and collecting duct [13,14,15]. Like MARCKS, spectrin is an actin cytoskeleton protein that associates with ENaC in renal epithelial cells [16].

Cathepsin B is known to regulate lipid metabolism by cleaving various proteins [4]. Cathepsin B knockdown was reported to reduce cellular triglycerides [4]. Another study showed cathepsin overexpression induces perilipin-1 degradation leading to lipid metabolism dysfunction in adipocytes [17]. Multiple bioactive lipid metabolites are produced during pathophysiological conditions and also during normal phospholipid turnover. Lysophosphatidylcholine (LPC) is a bioactive lipid metabolite formed by phospholipase A2-mediated hydrolysis of phosphatidylcholine [18]. Several biochemical studies have shown low concentrations of LPCs activate protein kinase C (PKC) in various cell types [19,20,21,22].

Although cathepsin B is one of the most abundantly expressed proteases in the kidney, and it has been shown to regulate the ENaC-MARCKS pathway at the apical plasma membrane [7,8], its role in regulating other effector proteins and lipids in the kidney is largely unknown. The diverse roles of cathepsin B coupled to a knowledge gap in the regulation of lipids and proteins by this protease in the native kidney led us to become interested in further investigating the role of cathepsin B in regulating membrane lipids and the activity of various kinases in the kidney.

Here, we investigated for the first time whether changes in the amount of bioactive membrane lipids in the kidney cortex of cathepsin B knockout mice correlate with changes in MARCKS protein expression and PKC activity. This study is significant at multiple levels. First, we confirm the role of cathepsin B in the proteolysis of MARCKS and spectrin proteins in the native kidney using a transgenic cathepsin B knockout mouse model. Second, we confirm a direct association between MARCKS and spectrin in the kidney that is attenuated in cathepsin B knockout mice. Third, we characterized the lipid profile of the kidney cortex from cathepsin B knockout mice compared to wild-type mice. Finally, we identified a decrease in protein kinase C activity in the kidney cortex of cathepsin B knockout mice compared to wild-type control mice. Collectively, these findings are important in the understanding of the diverse roles of cathepsin B in the kidney that may lead to the development of novel drug targets for various diseases including hypertension. 

## 2. Materials and Methods

### 2.1. Animals and Diet

All animal studies were approved by the University of Florida’s Institutional Animal Care and Use Committees. A total of 4 female and 3 male Cathepsin B knockout mice (stock No. 030971) (Jackson Laboratory; Bar Harbor, ME, USA), ten weeks of age, 1 female and 2 male 129Sv wild-type mice of the same age, and 2 females and 3 male C57B6 wild-type mice of the same age were maintained on a normal salt diet of 0.4% NaCl gel diet prepared from a powdered from (TEKLAD TD 94045) (Envigo; Madison, WI, USA) for the duration of the study. Water was provided ad lib. Table 1 shows the body and kidney weight of the mice. 

### 2.2. Tissue Lysate Preparation, BCA Assay and Western Blotting

A total of 10 mg of snap-frozen kidney cortex tissue from each mouse was washed with 1XPBS (prepared from a 10× solution (1.37 M NaCl, 100 mM Na_2_HPO_4_, 27 mM KCl, 18 mM KH_2_PO_4_, PH to 7.4) and then homogenized (Omni TH homogenizer; Warrenton, VA, USA) in 500 μL tissue protein extraction reagent (TPER) (ThermoFisher Scientific; Waltham, MA, USA). The tissue was centrifuged at 13,000 rpm for 10 min at 4 °C. Next, 450 µL of the resulting supernatant was subjected to ultracentrifugation at 34,000 rpm for 30 min at 4 °C. The resulting pellets were reconstituted in 200 µL TPER and then sonicated twice for 3 s intervals while on ice. Serial dilutions of a stock 1 mg/mL solution of BSA (Sigma-Aldrich, St. Louis, MO, USA) were performed to prepare 9 BSA standards. A 1:10 dilution of each tissue lysate sample was mixed with the BCA reagents (ThermoFisher Scientific), and the plate was read at 570 nm before calculating the protein concentration of each sample. A total of 50 μg of total protein were resolved on 4–20% Tris Glycine gels (ThermoFisher Scientific) at 200 volts for 60 min on a Criterion electrophoresis system (BioRad; Hercules, CA, USA). Proteins were electrically transferred onto nitrocellulose membranes (ThermoFisher Scientific) using a Criterion transfer system (BioRad) for 120 min in chilled Towbin buffer (5 mM Tris, 192 mM Glycine, 20% Methanol (*v*/*v*)). Using a 5% nonfat dry milk solution prepared in 1XTBS, the membranes were blocked for 1 h at room temperature. Next, each blot was incubated with a 1:1000 dilution of primary antibody (anti-MARCKS (ab72459; Abcam, Cambridge, MA, USA), anti-spectrin (PA1-25008; ThermoFisher Scientific). After a series of washes with 1XTBS (BioRad), the blots were incubated with a 1:3000 dilution of goat anti-rabbit secondary (BioRad) for 1 h at room temperature. Next, ECL Western blotting detection reagent (BioRad) was incubated with the blots for 5 min before imaging the blots on a ChemiDoc imaging system (BioRad). 

### 2.3. Immunoprecipitation Studies

A 1:250 dilution of anti-MARCKS polyclonal antibody was combined with 100 μg total tissue lysate from the kidney cortex of cathepsin B knockout mice, 129Sv wild-type mice, and C57B6 wild-type mice. After a 2–4 h incubation at 4 °C with end-over-end mixing, the resulting mixture was then incubated with a prewashed 50% protein A agarose slurry (ThermoFisher) at a 1:10 dilution at 4 °C for 4–6 h with end-over-end mixing. The beads were washed three times with ice-cold T-PER, and the resulting complexes were eluted in 1× SDS sample buffer. The eluted samples were analyzed by SDS-PAGE and Western blotting.

### 2.4. Lipid Extraction from Kidney Cortex Membrane Fractions

The Bligh and Dyer method was used to extract lipids from each sample [23], which was then adjusted to 1 mL with reagent-grade water and incubated for 10 min on ice. Subsequently, 2 mL of methanol and 0.9 mL of methylene chloride were added to the mixture that was vortexed for a duration of 30 s. A mixture of EquiSPLASH Lipidomix (Avanti Polar Lipids, Inc., Alabaster, AL, USA) consisting of 13 deuterated lipids at a concentration of 100 µg/mL was used as an internal standard. The deuterated lipids included 15:0–18:1(d7) diacylglycerol (DAG); 18:1(d7) monoacylglycerol (MAG); d18:1–18:1(d9) sphingomyelin (SM); C15 ceramide (CER)-d7; 15:0–18:1(d7)–15:0 triacylglycerol (TAG); 18:1(d7) cholesteryl ester (CE); 15:0–18:1(d7) phosphatidylcholine (PC); 18:1(d7) lysophosphatidylcholine (LPC); 15:0–18:1(d7) phosphatidylglycerol (PG); 15:0–18:1(d7) phosphatidylinositol (PI); 15:0–18:1(d7) phosphatidylethanolamine (PE); 18:1(d7) lysophosphatidylethanolamine (LPE); and 15:0–18:1(d7) phosphatidylserine (PS). Dilutions of 1:5 were made of the internal standards and a total of 50 μL (20 μg/mL for each standard) was added to each sample and incubated for a duration of 30 min at room temperature. Thereafter, 0.9 mL of methylene chloride and 1 mL of reagent-grade water were mixed by inversion 10 times, followed by centrifugation at 100× *g* for 10 min. After a second round of extraction was performed with 2 mL of methylene chloride, the pooled organic phases were dried with N2, resuspended in 50 µL of 96% ethanol, and analyzed using liquid chromatography—mass spectrometry.

### 2.5. LC-MS/MS Conditions

An ultra-high-performance chromatography (UHPLC) system (Shimadzu Co., Columbia, Maryland) that was connected with a QTrap 6500 mass spectrometer (AB Sciex, Redwood City, CA, USA) permitted detailed lipid samples analysis. Chromatographic separation was conducted with an XBridge Amide 3.5 µm, 4.6 × 150 mm column (Waters Corp, Milford, MA). A dual gradient of acetonitrile and water with a ratio of 95:5 (*v*/*v*) for mobile phase A and B 50:50 (*v*/*v*) was applied. In each solvent, 1 mmol/L ammonium acetate was present with an adjusted pH of 8.2 in the freshly produced mobile phases. The linear gradient of solvent B increased to 6% within 6 min and reached 25% within 4 min, 98% within 1 min and 100% within 2 min with a flow rate of 0.7 mL·min^−1^. The column was flushed using mobile phase B at a flow rate of 1.5 mL·min^−1^ for 3 min and 5 μL of the samples was injected into the UHPLC before the effluent was transferred to the ion source using nanoViper fittings and tubing with a 100 µmol/L ID (nanoViper capillary IDXL, ThermoFisher Scientific). The schedule MRM mode was utilized during mass spectrometry procedures, which included incorporated negative and positive ion modes.

The electrospray ionization source settings included the declustering potential for the positive mode adjusted to 60 and the negative mode adjusted to 80. The distinct lipid species located in the positive and negative modes received varying collision energy levels from 25 to 60. The entrance potential was fixed at 10 and the collision cell exit potential was fixed at 15. The ion spray voltage did not deviate from the programmed 4.5 kV and its temperature was set at 300 °C. For the inclusion of technical replicates, each sample was injected twice. To avoid cross-contamination, three techniques were performed, which included washing the column and tubing with a high flow rate of mobile phase B, an additional wash of the needle with 500 µL with isopropanol, and finally administering blanks throughout the procedure at determined set intervals.

### 2.6. Lipid Quantification 

Using the Analyst software (ver. 1.6.2), the study generated a comprehensive data list of 19 different types of lipids, which included PC, PE, PI, PG, PS, LPC, LPE, lysophosphatidylinositol (LPI), lysophosphatidylglycerol (LPG), lysophosphatidylserine (LPS), TAG, DAG, MAG, CE, SM, CER, dihydroceramide (DCER), hexosylceramide (HCER), and lactosylceramide (LCER). The peaks were not subjected to Gaussian smoothing during the inspection process. Through the utilization of MultiQuant software (ver. 3.0.3) and in conjunction with the concentration of the related internal standard, the relative quantity of each lipid class was computed. The instruments performance was verified through the utilization of a standard lipid profile from bovine heart extract. The values were calculated as the mean of the samples in each group, which were then normalized based on equal protein concentration between the two groups. 

### 2.7. PKC Kinase Activity Assay

A PKC activity assay (Table 2) was performed according to the manufactures instructions in order to calculate relative PKC activity in C57B6 and CBN tissue lysates.

### 2.8. CAMKII Beta Assay

A CAMKII Beta assay (Table 2) was performed according to the manufactures instructions in order to calculate the activity of CAMKII Beta activity in C57B6 and CBN tissue lysates.

### 2.9. Phosopholipase D Assay

A Phospholipase D assay (Table 2) was performed according to the manufactures instructions in order to calculate the activity of phospholipase D activity in C57B6 and CBN tissue lysates.

### 2.10. Data Analysis and Statistics

All results are presented as mean ± SEM. Statistical differences between the two groups were determined after performing a Student *t*-test in SigmaPlot software (Jandel Scientific, San Rafael, CA, USA). Differences with a *p*-value of <0.05 were considered significant.

## 3. Results

### 3.1. Reduced MARCKS Protein Expression in Cathepsin B Knockout Mice Compared to C57B6 Wild-Type Mice 

**Figure 1:**
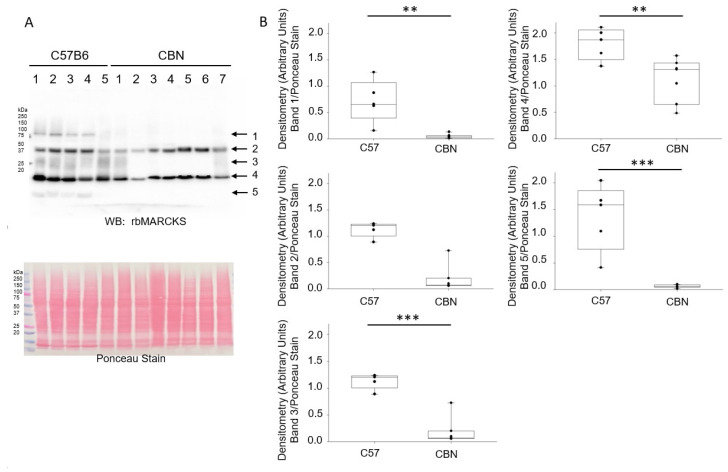
Western blot and densitometric analysis of MARCKS protein in kidney cortex lysates from C57B6 wild-type C57B6 mice and Cathepsin B knockout (CBN) mice. (**A**) Western blot (top) of unprocessed and cleaved forms of MARCKS using a recombinant rabbit polyclonal antibody. The corresponding Ponceau stain that was used to assess lane loading is shown (bottom). (**B**) Densitometric analysis of the unprocessed and proteolytically cleaved forms of MARCKS for the immunoreactive bands indicated by arrows in panel (**A**). *N* = 5 C57B6 mice and *N* = 7 CBN mice. ** represents a *p*-value < 0.01, *** represents a *p*-value < 0.001.

Although the association between MARCKS and the inner leaflet of the plasma membrane is thought to be regulated by cathepsin B, the expression and proteolysis of MARCKS protein in kidney membranes of cathepsin B knockout mice has not yet been investigated. Here, we investigated protein expression and proteolysis of MARCKS protein in kidney cortex membrane fractions of cathepsin B knockout mice compared to aged matched C57B6 wild-type mice. As shown in Figure 1, Western blot and densitometric analyses show there is less abundance of the unprocessed and cleaved forms of MARCKS in the kidney of cathepsin B knockout mice compared to wild-type mice.

### 3.2. Decrease Abundance of the Actin Cytoskeleton Protein Spectrin in Kidney Cortex Lysates of Cathepsin B Knockout Mice

**Figure 2:**
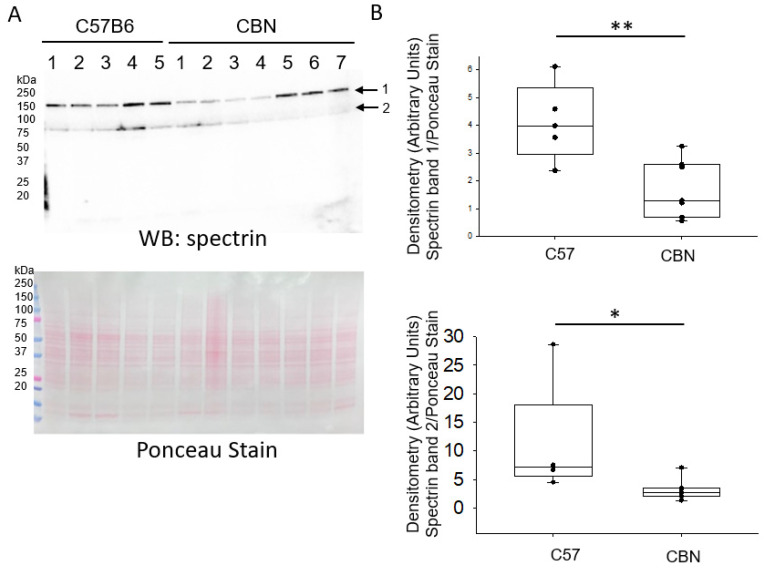
Western blot and densitometric analysis of spectrin protein in kidney cortex lysates from C57B6 wild-type mice and Cathepsin B knockout (CBN) mice. (**A**) Western blot (top) of unprocessed and cleaved form of spectrin using a commercial polyclonal antibody. The corresponding Ponceau stain that was used to assess lane loading is shown (bottom). (**B**) Densitometric analysis of the unprocessed and proteolytically cleaved form of spectrin for the immunoreactive bands indicated by arrows in panel (**A**). *N* = 5 C57B6 mice and *N* = 7 CBN mice. *, represents a *p*-value < 0.05, ** represents a *p*-value < 0.01.

Next, we examined whether there is less abundance and proteolysis of spectrin in the kidney cortex of cathepsin B knockout mice. As shown by the Western blot and densitometric analysis in Figure 2, similar to MARCKS protein, there was less abundance and proteolysis of spectrin protein in kidneys of mice lacking cathepsin B compared to C57B6 wild-type control mice. 

### 3.3. Interaction between MARCKS and Spectrin Proteins in the Kidney

**Figure 3:**
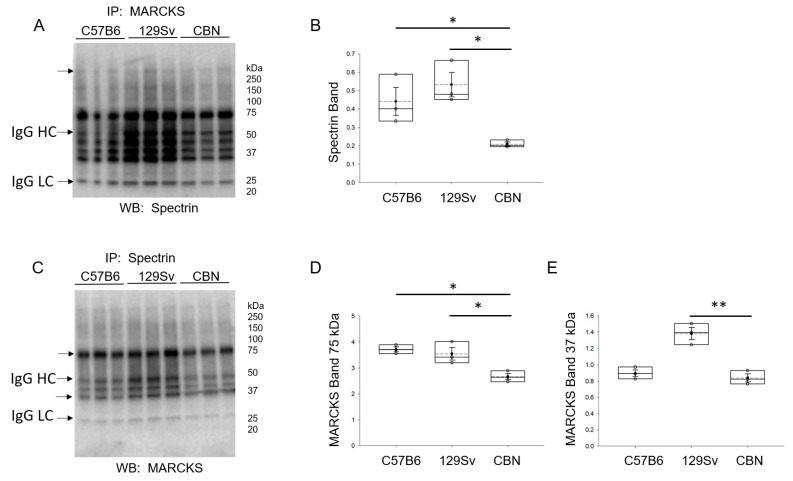
Interaction of MARCKS and spectrin proteins in the kidney of cathepsin B knockout mice compared to C57B6 wild-type mice and 129Sv wild-type mice. (**A**) Western blot for Spectrin protein after immunoprecipitating MARCKS protein from kidney cortex lysates of cathepsin B knockout mice compared to C57B6 wild-type mice and 129Sv wild-type mice. (**B**) Densitometric analysis of the immunoreactive >250 band corresponding to spectrin protein. (**C**) Western blot for MARCKS protein after immunoprecipitating spectrin protein from kidney cortex lysates of cathepsin B knockout mice compared to C57B6 wild-type mice and 129Sv wild-type mice. (**D**) Densitometric analysis of the immunoreactive 75 kDa band corresponding to MARCKS protein. (**E**) Densitometric analysis of the immunoreactive 37 kDa band corresponding to the cleaved form of MARCKS protein. *N* = 3 mice in each group. * represents a *p*-value < 0.05, ** represents a *p*-value < 0.01. Arrows indicate the immunoreactive bands used for densitometric analysis. IgG HC represents the heavy chain of IgG. IgG LC represents the light chain of IgG. The immunoreactive IgG LC bands in each blot were used for normalization. A secondary antibody only control experiment was performed (Supplementary Figure S1).

We performed immunoprecipitation Western blot studies to assess differences in the interaction between MARCKS and Spectrin protein in the kidney of cathepsin B knockout mice compared to C57B6 wild-type mice and 129Sv wild-type mice. As shown in Figure 3, there is less interaction between MARCKS and spectrin proteins in the kidney cortex of cathepsin B knockout mice compared to either wild-type group. 

### 3.4. Differential Expression of Lipids in Kidney Cortex Membrane Fractions of C57B6 Wild-Type and Cathepsin B Knockout (CBN) Mice

**Figure 4:**
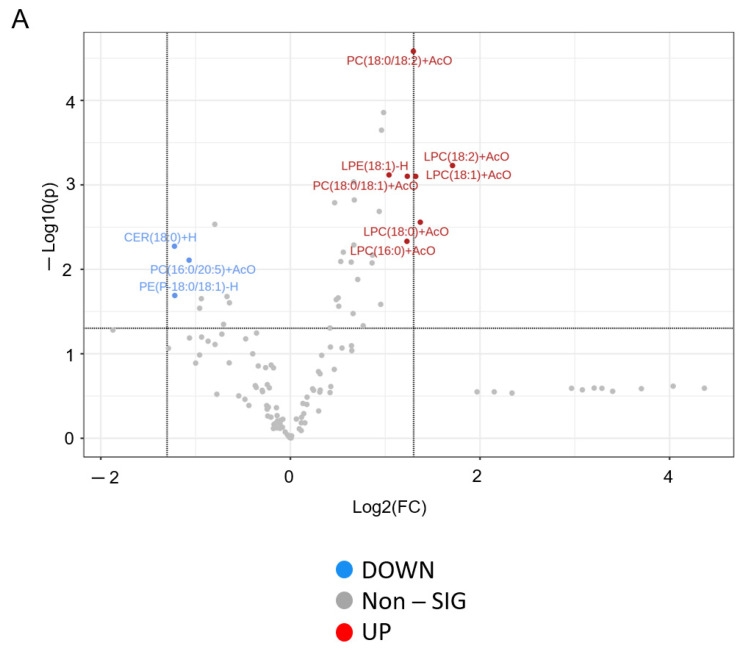

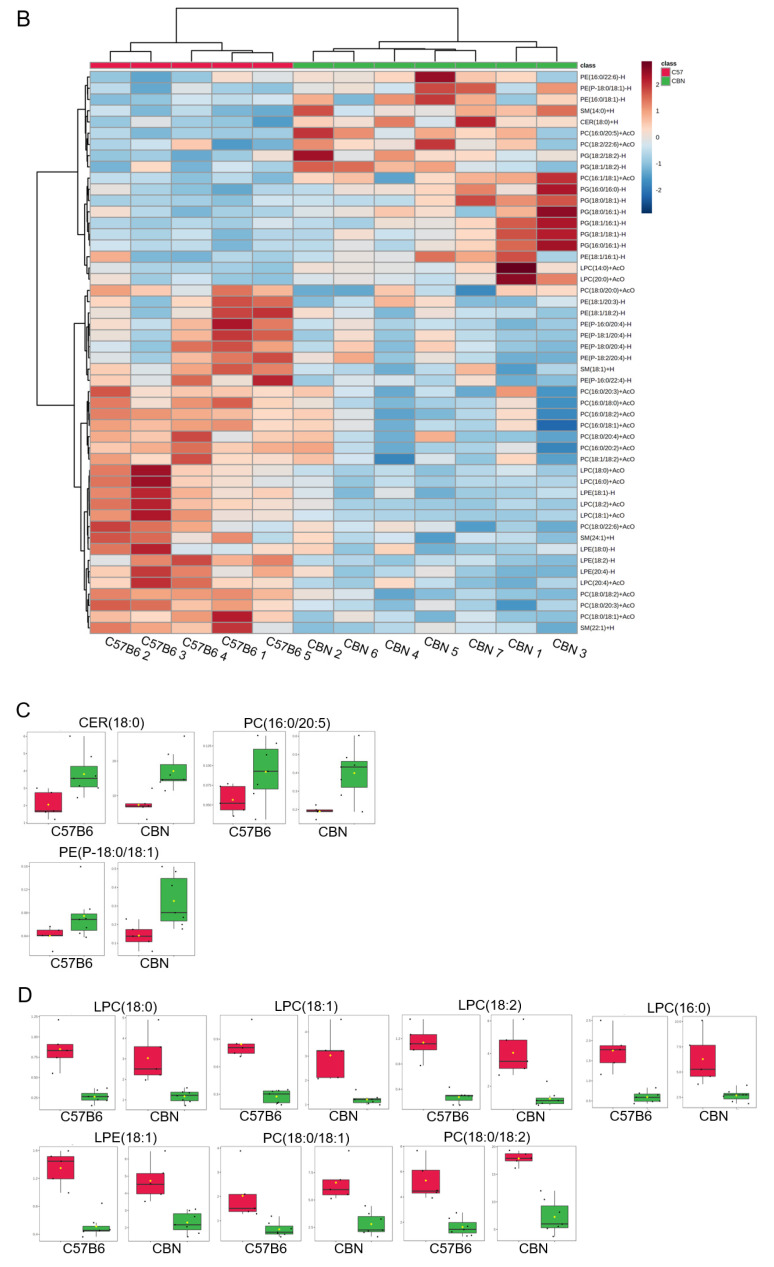
Lipidomic analysis of lipids between kidney cortex membrane fractions of C57B6 wild-type and Cathpesin B knockout (CBN) mice. (**A**) Volcano plot showing all lipids including those with a fold change threshold of 2.0 and a *p*-value of < 0.01. (**B**) Heatmap of the top 50 lipids with a focus on patterns from important features. (**C**) Lipids significantly down regulated. (**D**) Lipids significantly up-regulated. Plots of original concentrations (left) and plots of normalized concentration (right). *N* = 5 C57B6 mice and *N* = 7 cathepsin B knockout mice.

Cathepsin B is known to regulate a myriad of proteins including proteins involved in lipid metabolism [4,17]. However, changes in the kidney membrane lipid composition have not been investigated in cathepsin B knockout mice. In this study, lipids were extracted from kidney cortex membrane fractions from cathepsin B knockout mice and aged matched C57B6 wild-type mice. Mass spectrometry-based lipidomics was performed to investigate changes in lipid classes and species between membrane fractions from the two groups. A volcano plot and heat map of differences in lipid abundance between kidney cortex membrane fractions of cathepsin B knockout and wild-type mice is shown in Figure 4A,B. The relative amount of Cer(18:0), PC(16:0/20:5), and PE(P-18:0/18:1) was greater in kidney cortex membranes of cathepsin B knockout mice compared to wild-type mice (Figure 4C). Conversely, the amounts of LPC(18:0), LPC(18:1), LPC(18:2), LPC(16:0), LPE(18:1), PC(18:0/18:1), and PC(18:0/18:2) were less in the kidney cortex membranes of cathepsin B knockout mice compared to wild-type mice (Figure 4D). 

### 3.5. Basal Activity of Proteases and Enzymes in the Kidney Cortex of Cathepsin B Knockout and C57B6 Wild-Type Mice

**Figure 5:**
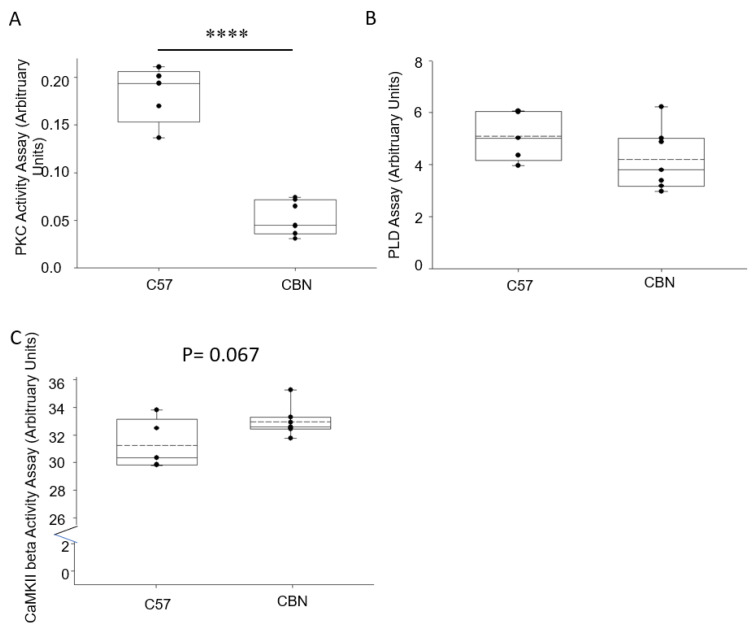
Endogenous enzymatic activity in kidney cortex lysate from C57B6 wild-type mice and Cathepsin B null mice. (**A**) Basal PKC activity in kidney cortex tissue lysates. (**B**) Basal PLD activity in kidney cortex tissue lysates. (**C**) Basal CaMKII activity in kidney cortex tissue lysates. **** represents a *p*-value < 0.0001.

Since various lipids play an important role in regulating the activity of different kinases and enzymes, we next investigated changes in the basal activity of specific proteins in the kidney cortex that are known to regulate actin cytoskeleton proteins. PKC activity was markedly reduced in kidney lysates from cathepsin B knockout mice compared to wildtype mice (Figure 5). However, PLD activity and CaMKII activity was comparable between the two groups (Figure 5B,C).

## 4. Discussion

The MARCKS family of proteins play an important role in the organization of the actin cytoskeleton. Posttranslational modifications including myristoylation, phosphorylation, and proteolysis affect the subcellular localization of MARCKS in renal epithelial cells. In distal tubule and collecting duct cells, calpain dependent cleavage at a site after the effector domain of MARCKS is thought to maintain the hydrophobic and electrostatic association between the protein at the plasma membrane by preventing PKC to access serine residues within the effector domain of the protein [24]. However, other proteases such as cathepsins cleave various sites of MARCKS. Seminal studies by Spizz and Blackshear showed that proteolysis of MARCKS results in stable amino terminal and carboxyl terminal fragments [25]. Subsequent studies by this group demonstrated cathepsin B largely contributes to the proteolysis of MARCKS and the cathepsin B-mediated cleavage occurs within the phosphorylation site domain of MARCKS [12]. Interestingly, it was suggested that MARCKS may regulate the secretion and/or action of cathepsin B [12].

Spectrin is another actin cytoskeleton protein that is regulated by proteolysis [26]. Cho et al. showed the cell-permeable methyl ester derivative of the negatively charged cathepsin B inhibitor CA074ME reduces α-spectrin cleavage, similar to the effect of calpain inhibition [27]. Moreover, Taylor et al. showed proteolysis of spectrin in erythrocytes results in the biogenesis of extracellular vesicles from the plasma membrane [28]. 

Although our lipidomic studies identified changes in multiple lipid species including Ceramides (Cer), phosphatidylcholines (PC), phosphatidylethanolamines (PE), lysophosphatidylethanolamines (LPE), and lysophosphatidylcholines (LPC) between cathepsin B knockout mice compared to wild-type mice, we focused on investigating the physiological significance of the observed reduction in LPCs in membrane fractions of the kidney cortex. The reason for this is because LPC is an important bioactive lipid that is associated with the generation of chemo-attractants and reactive oxygen species (ROS), and it has been previously shown to contribute to the rapid progression of diabetic kidney disease [29]. A different study showed that intravenous infusion of myristoyl–lysophosphatidylcholine reduces systemic blood pressure and renal plasma flow and results in increased excretion of sodium and water in male Sprague Dawley rats [30]. Importantly, multiple studies have demonstrated LPC activates PKC in mesangial cells [31,32] and endothelial cells [33]. Another study showed LPC treatment induces phosphorylation of PKCζ in human melanocytes [34].

**Figure 6:**
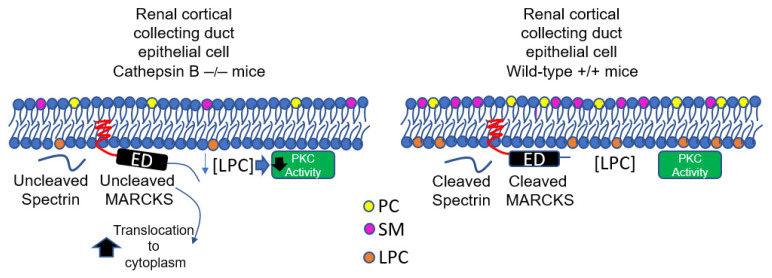
Proposed model. Lysophosphatidylcholine (LPC) is produced by the phospholipase A(2)-mediated hydrolysis of phosphatidylcholine. There is less expression and proteolysis of the actin cytoskeleton proteins MARCKS and spectrin, and lower PKC activity in cathepsin B knockout mice compared to wild-type mice.

As shown in Figure 6, our results of cathepsin B knockout mice having significantly lower PKC activity in the native kidney compared to wild-type mice is not surprising since other groups have indicated cathepsin B downregulation may result in lower PKC expression. Alapati et al. showed shRNA-mediated down-regulation of uPAR and cathepsin B resulted in reduced expression levels of multiple PKC isoforms in glioma-initiating cells [35]. 

The lipidomic profiles and decrease in PKC activity in the kidney of cathepsin B knockout mice that are presented in this study contribute to the general understanding of MARCKS regulation. However, this study does have some limitations. One limitation of this study is that we did not investigate whether the decrease in LPC concentrations in the kidney membranes of cathepsin B knockout mice compared to wild-type mice contributes to the decrease in PKC activity. A second limitation of our study is that we did not investigate whether the absence of cathepsin B leads to changes in extracellular vesicle biogenesis and release. This is conceivable since our group previously demonstrated that this protease is enriched in exosomes [9]. Finally, we did not investigate regional differences in actin cytoskeleton proteins, lipids, kinases, and enzymes in the kidney cortex and medulla of cathepsin B knockout mice and wild-type mice. Since studies have shown regional differences in various lipids and proteins in the kidney, studies aimed at examining regional differences between cathepsin B and wild-type mice are warranted. Additionally, previous studies by our group investigated differences in lipid profiles from renal cortex tissues from diabetic db/db mice treated with vehicle or dapagliflozin [36] and from non-nephrotic mice and nephritic mice [37]. These data suggest there are differences in a myriad of lipid classes and species between mice of different genetic backgrounds. However, this is the first study to investigate changes in the lipid profiles within the kidney cortex of cathepsin B knockout mice. Since the cathepsin B knockout mice are on a mixed genetic background, comparisons of lipid profiles in the kidney cortex of these mice, with mice of a pure genetic background are not straightforward.

In addition to regulating the function of the actin cytoskeleton proteins MARCKS and spectrin in healthy renal epithelial cells, cathepsin B has been studied in cancer and its therapeutic potential has been investigated. A previous study by Gonzalez et al. demonstrated a cathepsin B cleavable linker can be used in the release of a desired prodrug for the treatment of prostate cancer cells [38]. Similarly, Debnath et al. used a cleavable design strategy for the desirable controlled release of a prodrug in cancer therapy [39].

Taken together, our results suggest the absence of cathepsin B in the kidney, at least in part, contributes to the decrease in MARCKS and spectrin proteolysis, the inhibition of PKC activity, and the decrease in LPC within kidney membranes.

## Figures and Tables

**Table 1:** Measurements of body weight and kidney weight from CathB −/− knockout mice, 129Sv wild-type mice, and C57B6 wild-type mice. Weights are in grams and presented as mean +/− SEM.

Parameter	CBN	129Sv Wild-Type	C57B6 Wild-Type
Body Weight	26.14 +/− 1.76	26.33 +/− 1.8	26.6 +/− 1.5
Kidney Weight	0.26 +/− 0.01	0.27 +/− 0.01	0.26 +/− 0.02

**Table 2:** Assays used in this study.

Assays	Manufacture	Cat. No.
CAMKII Beta Assay	My Biosource	MBS456030
Phsopholipase D Assay Kit PKC Kinase Activity Assay Kit	Sigma Aldrich Abcam	MAK-137 ab139437

## Data Availability

The individual data points of each dataset are shown within the plots.

## Supplementary Materials

The following supporting information can be downloaded at: https://www.mdpi.com/article/10.3390/biomedicines11051489/s1, Figure S1, Secondary Antibody only control.
