# Supplementary figures and images for: Decreased MARCKS Protein Expression in Kidney Cortex Membrane Fractions of Cathepsin B Knockout Mice Is Associated with Reduced Lysophosphatidylcholine and Protein Kinase C Activity
Source: Biomedicines. 2023 May 20;11(5):1489. doi: 10.3390/biomedicines11051489 (PMC10216610; doi:10.3390/biomedicines11051489)

## Slide 1
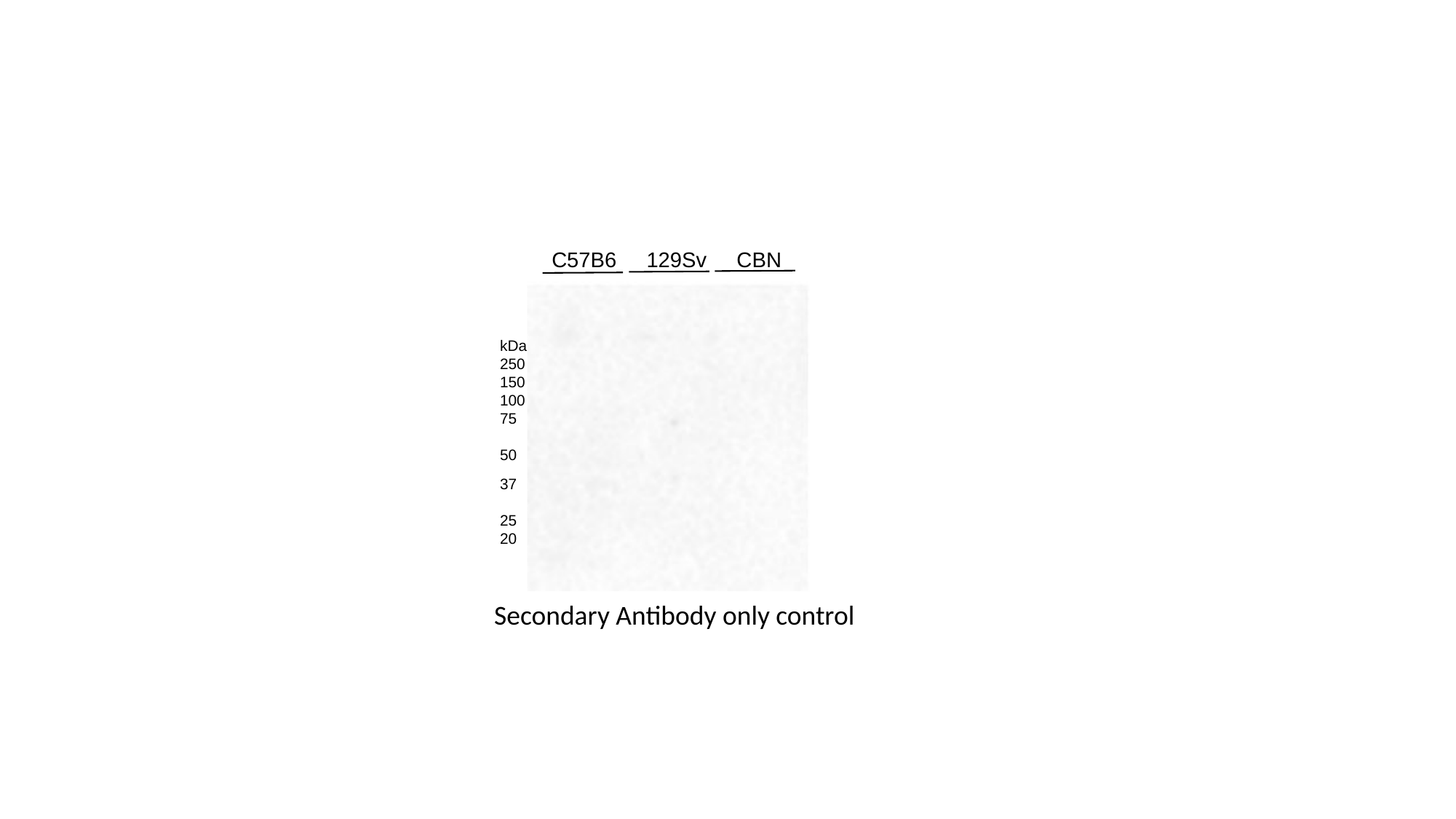

C57B6 129Sv CBN
kDa
250
150
100
75
50
37
25
20
Secondary Antibody only control

Supplement: Supplementary file 1 [file biomedicines-11-01489-s001.zip › supfig1.pptx]
